# Optimizing GLP-1 therapies for obesity and diabetes management

**DOI:** 10.1016/j.obpill.2025.100222

**Published:** 2025-10-24

**Authors:** Jarvis C. Noronha, Luc F. Van Gaal, Ian J. Neeland, Angela Fitch, Andreas FH. Pfeiffer, Laura Chiavaroli, Cyril WC. Kendall, John L. Sievenpiper

**Affiliations:** a Toronto 3D Knowledge Synthesis and Clinical Trials Unit, Clinical Nutrition and Risk Factor Modification Centre, St. Michael's Hospital, Toronto, Ontario, Canada; b School of Medicine, Faculty of Medicine, The University of Queensland, Brisbane, Queensland, Australia; c Department of Endocrinology, Diabetology and Metabolism, Antwerp University Hospital, University of Antwerp, Antwerp, Belgium; d Department of Medicine, Case Western Reserve University School of Medicine, Cleveland, OH, USA; e Division of Cardiovascular Medicine, Harrington Heart and Vascular Institute, University Hospitals Cleveland, Cleveland, OH, USA; f knownwell, Boston, MA, USA; g Department of Endocrinology and Metabolic Diseases, Charité Universitätsmedizin Berlin, Campus Benjamin Franklin, Berlin, Germany; h Deutsches Zentrum für Diabetesforschung e.V., Helmholtz Center Munich, Neuherberg, Germany; i Department of Nutritional Sciences, Temerty Faculty of Medicine, University of Toronto, Toronto, Ontario, Canada; j Li Ka Shing Knowledge Institute, St. Michael's Hospital, Toronto, Ontario, Canada; k College of Pharmacy and Nutrition, University of Saskatchewan, Saskatoon, Saskatchewan, Canada; l Department of Medicine, Temerty Faculty of Medicine, University of Toronto, Toronto, Ontario, Canada; m Division of Endocrinology and Metabolism, Department of Medicine, St. Michael's Hospital, Toronto, Ontario, Canada

**Keywords:** GLP-1 therapies, Semaglutide, Liraglutide, Tirzepatide, Pharmacotherapy, Nutrition therapy, Lifestyle intervention, Lean body mass, Obesity management, Diabetes management, Weight maintenance, Gastrointestinal side effects

## Abstract

**Background:**

Glucagon-like peptide-1 (GLP-1) therapies are highly effective for weight loss and metabolic improvement in obesity and type 2 diabetes management. However, their use poses clinical challenges, including loss of lean muscle mass and gastrointestinal side effects, both of which may affect adherence and long-term outcomes.

**Methods:**

This commentary synthesizes current evidence and expert perspectives, drawing on presentations from the 42nd International Symposium on Diabetes and Nutrition by the Diabetes and Nutrition Study Group, to develop practical recommendations for integrating nutrition and physical activity with GLP-1 therapies for obesity and diabetes management.

**Results:**

We summarize consensus recommendations from a global working group, organized into seven thematic modules, to guide alignment of GLP-1 therapies with dietary and lifestyle interventions across the key stages of the weight management journey. Evidence from several clinical trials demonstrate that the combination of GLP-1 therapies with structured dietary and exercise interventions results in additive weight loss effects compared with either strategy alone. Strategies to preserve lean mass with GLP-1 therapies include achieving protein intakes >1.2 g/kg/day, evenly distributed across meals, combined with aerobic activity and structured resistance training. Specific recommendations are provided to minimize nausea, vomiting, diarrhea, and constipation associated with GLP-1 therapies, as well as to prevent and manage complications such as, cholelithiasis and gastroesophageal reflux disease. Future research priorities include examining the impact of GLP-1 therapies on dietary habits and physical activity levels, improving muscle health assessment, and testing pharmacologic adjuncts to limit lean mass loss.

**Conclusion:**

Maximizing the benefits of GLP-1 therapies require a multidisciplinary approach that integrates evidence-based nutrition, physical activity, and proactive management of gastrointestinal side effects. Such an approach can enhance adherence, preserve functional capacity, and sustain the long-term benefits of these therapies.

## Graphical abstract

**Figure:**
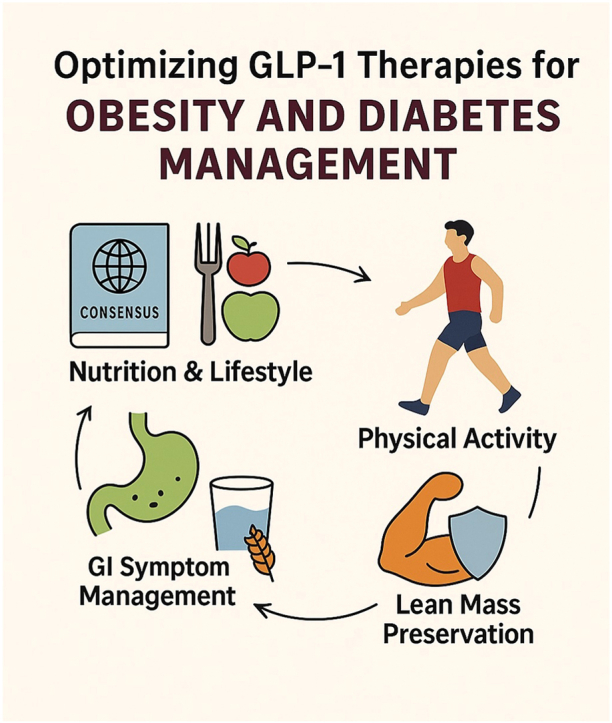

## 1 Introduction

Glucagon-like peptide-1 (GLP-1) therapies, including liraglutide and semaglutide, have emerged as key treatments for obesity and type 2 diabetes management [1,2]. They mimic the incretin hormone GLP-1 to improve glucose-dependent insulin secretion, delay gastric emptying, and reduce appetite, leading to significant weight loss and metabolic improvements [3]. However, their use raises important clinical considerations. Because long-term efficacy depends not only on pharmacologic effects but also on maintaining healthy dietary patterns and regular physical activity, as demonstrated in the landmark trials, attention to lifestyle factors is important when prescribing these agents [[4], [5], [6]]. In addition, GLP-1 therapies affect body composition by reducing not only fat mass but also lean mass, a concern that is particularly relevant for at-risk groups such as older patients and those with sarcopenic obesity [[7], [8], [9]]. Gastrointestinal (GI) side effects are also common in GLP-1 therapies and are a leading cause of treatment discontinuation [[10], [11], [12]]. Thus, optimizing GLP-1 therapies requires an integrated approach that supports adequate nutrition and physical activity, addresses body composition changes, and proactively manages GI side effects to maximize adherence and long-term clinical benefit.

In this commentary, we synthesize current evidence and expert perspectives, drawing on presentations from the 42nd International Symposium on Diabetes and Nutrition by the Diabetes and Nutrition Study Group, to provide practical guidance on optimizing the use of GLP-1 therapies in obesity and diabetes management. We first present summaries of consensus recommendations from an international working group that combine nutrition and lifestyle interventions with GLP-1 therapies followed by examining the role of diet and physical activity in maximizing weight loss. Strategies to preserve lean mass are discussed, followed by approaches to prevent and manage GI side effects and complications to support treatment adherence. We conclude by outlining key research gaps and future priorities.

## 2 Integrating nutrition and physical activity with GLP-1 therapies for obesity and diabetes management: an international consensus

**Fig. 1:**
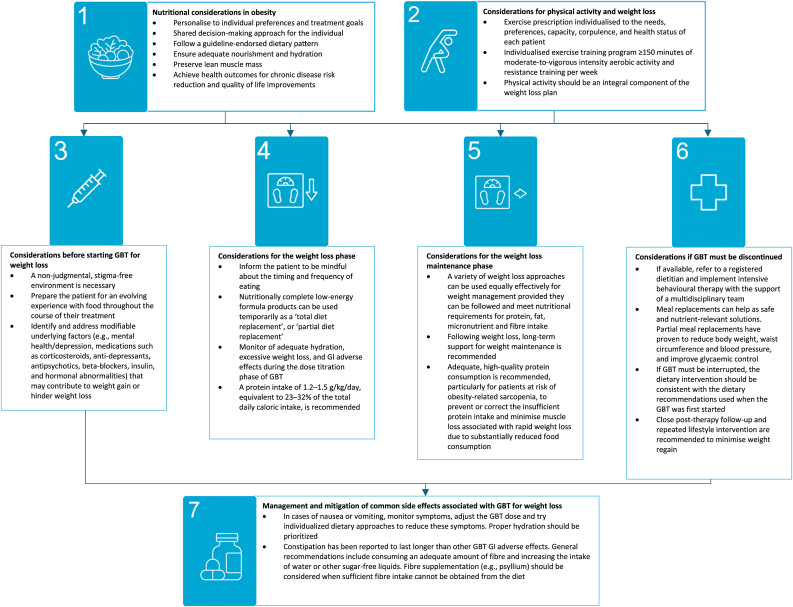
Recommendations to optimize GLP-1 therapies for the management of obesity and diabetes.

Fig. 1 summarizes consensus statements from a global working group on nutritional and lifestyle recommendations for managing obesity and diabetes with GLP-1 therapies. These statements were developed by an international panel of 15 expert clinicians and nutritionists using a modified Delphi process conducted from May to September 2024. The process included a comprehensive literature review (from January 2021–March 2024), panel discussions, and two rounds of rating. Consensus was defined as ≥67 % agreement (≥10 of 15 experts) with a score of ≥7 on a 9-point scale, with each expert's input equally weighted at 7 %. The review drew on high-quality evidence from clinical practice guidelines, systematic reviews, randomized and non-randomized trials, and cohort studies. Final statements were grouped into seven thematic modules, reflecting key stages of the weight management journey.

## 3 Combining GLP-1 therapies with diet and exercise – are the weight loss effects additive?

The pivotal clinical trials of GLP-1 therapies were conducted in conjunction with structured, guideline-recommended lifestyle inventions which comprised the standard of care for weight loss interventions. For example, in the STEP 1 (Semaglutide Treatment Effect in People with Obesity) trial, all participants received individual counseling every four weeks to support adherence to a reduced-calorie diet (500 kcal/day deficit from estimated energy expenditure) and to encourage at least 150 minutes per week of physical activity, such as walking. Diet and physical activity were recorded daily and reviewed during counseling sessions [4]. At week 68, mean weight change among participants was −14.9 % in the semaglutide group vs −2.4 % with placebo. Similarly, in the SURMOUNT-1 trial, all participants received lifestyle intervention which included regular counseling with a dietitian or qualified health professional to achieve a 500 kcal/day energy deficit and ≥150 minutes of physical activity weekly. At week 72, mean weight loss was −15.0 % (5 mg tirzepatide), −19.5 % (10 mg tirzepatide), and −20.9 % (15 mg tirzepatide) vs −3.1 % with placebo [5]. These study designs illustrate that the weight loss outcomes reported in these trials reflect a pharmacologic effect in addition to structured lifestyle support.

Additional insights come from the phase 3 STEP trial (STEP 3), which combined 2.4 mg semaglutide with an intensive low-calorie diet (1000–1200 kcal/day meal replacements for the first 8 weeks, followed by a hypocaloric diet of 1200–1800 kcal/day) and progressive increases in physical activity to 200 minutes per week [13]. Both groups lost weight early, but separation emerged between weeks 4–8. Even under identical caloric prescriptions, semaglutide produced a −16.0 % weight loss by week 68 compared to −8.0 % with placebo, further supporting the additive pharmacologic effects of GLP-1 therapies beyond dietary and physical activity adherence.

The S-LiTE (Synergy effect of the appetite hormone GLP-1 [liraglutide] and Exercise on maintenance of weight loss and health after a low-calorie diet) trial provides perhaps the most rigorous test of additivity [6]. In this randomized, double-blind, placebo-controlled study, 215 individuals with obesity underwent an initial 8-week very low-calorie diet (800 kcal/day), followed by 52 weeks of maintenance with 3.0 mg liraglutide, structured exercise, both combined, or placebo. The exercise arm involved supervised group sessions with heart rate monitoring. At one year, compared with placebo, weight loss was −4.1 kg in the exercise group, −6.8 kg in the liraglutide group, and −9.5 kg in the combination group, highlighting the additive benefits of combining pharmacotherapy with exercise [6]. Beyond weight loss, the S-LiTE trial also evaluated body fat distribution and inflammatory markers [14]. Android fat percentage, a surrogate for visceral adiposity, was reduced in all active intervention arms, with the largest reduction in the combination group compared with exercise alone or liraglutide alone. These reductions were accompanied by decreases in high-sensitivity C-reactive protein (hs-CRP), with the combination group showing the greatest improvement compared with smaller reductions in the exercise and liraglutide groups [14]. Together, these findings reinforce that liraglutide and exercise not only have additive effects on weight loss but also on reducing visceral adiposity and systemic inflammation.

Long-term follow-up to week 104 in the S-LiTE trial demonstrated differing “legacy” effects. After stopping interventions at week 52, all groups regained weight, but exercise-containing groups regained less. From randomization to week 104, weight regain was +7.6 kg in placebo, +8.7 kg in liraglutide, +5.4 kg in exercise, and only +3.5 kg in the combination arm [15]. This suggests that exercise may confer some sustained benefit even after discontinuation, whereas pharmacotherapy benefits diminish rapidly when stopped.

An interesting aspect of the S-LiTE trial post-treatment follow-up was its examination of physical activity patterns. One year after treatment cessation, participants in the exercise-alone and exercise-liraglutide groups reported the highest levels of moderate-to-vigorous physical activity (MVPA), with median self-reported values of 240 and 225 minutes/week, respectively, compared to 150 minutes/week in placebo and only 30 minutes/week in the liraglutide-alone group [15]. A greater proportion of participants receiving liraglutide without exercise reported no structured activity, which corresponded to more daily sitting time (median 480 minutes/day) compared to the exercise-alone (420 minutes/day) and combination groups (420 minutes/day) [15]. Accelerometer measurements supported these findings, with MVPA of 134 minutes/day in the exercise group and 105 minutes/day in the combination group, versus 95 minutes/day for placebo and 88 minutes/day for liraglutide alone [15]. These data raise the hypothesis that patients on GLP-1 therapies may experience reduced physical activity, possibly due to inadequate energy intake (“not enough fuel in the tank”) or central effects on motivation. This highlights the importance of counseling patients not only on structured exercise but also on maintaining overall activity levels and minimizing sedentary behavior.

Dietary intake patterns may also shift with GLP-1 therapies. Across ten studies of GLP-1 receptor agonists and dual glucose-dependent insulinotropic polypeptide (GIP) agonists, caloric intake was reduced by approximately 16–39 % compared to placebo [16]. Most of these studies assessed intake using ad libitum test meals rather than 24-h dietary recalls, a methodological approach that offers the advantage of capturing voluntary eating behavior under controlled conditions but may underestimate total daily energy intake and limit extrapolation to real-world dietary patterns. Only one study reported providing dietary counseling alongside pharmacotherapy, and just two described involvement of a registered dietitian, underscoring a gap in structured nutritional guidance. This lack of formal nutritional support limits the ability to interpret how macronutrient composition, protein intake, and food selection are affected and underscores the need for future studies that integrate dietary counseling and standardized nutrition protocols. While four studies assessed macronutrient composition, results were inconsistent, leaving unanswered questions about whether GLP-1 induced reductions in dietary protein or other nutrients contribute to observed changes in body composition. Survey data from consumer populations suggest that individuals on GLP-1 therapies consume 700–800 fewer kilocalories per day than non-users, and tend to reduce intake of processed foods, sugar-sweetened beverages, refined grains, and beef [17]. However, the long-term effects of these changes on diet quality, micronutrient adequacy, and body composition remain largely unexplored.

Taken together, evidence from the STEP, SURMOUNT, and S-LiTE trials strongly supports the lifestyle interventions, particularly dietary modification and structured exercise, being not only compatible with but also additive to the effects of GLP-1 therapies. The combination of diet, exercise and GLP-1 therapies enhances weight loss, improves body composition, reduces visceral fat and inflammation, and may confer longer-term benefits in slowing weight regain. Additionally, sustained engagement in physical activity after treatment cessation appears more likely when exercise is integrated alongside pharmacotherapy, while GLP-1 therapies alone may be associated with lower post-treatment activity levels. Moreover, GLP-1 therapies appear to not only reduce caloric intake but also shift dietary patterns toward lower consumption of processed and energy-dense foods, suggesting potential qualitative as well as quantitative improvements in diet.

## 4 Preserving lean body mass during GLP-1 therapies with diet and exercise

While GLP-1 therapies are highly effective at producing substantial weight loss and metabolic improvements, there is concern regarding their impact on muscle quantity, health, and function [18]. Sarcopenia is a progressive, generalized disorder of skeletal muscle characterized by accelerated declines in muscle mass and function [19] and is associated with increased risk of frailty, falls, functional decline and mortality [20,21]. A recent systematic review and network meta-analysis of 22 randomized controlled trials involving 2258 participants evaluated the effects of GLP-1 receptor agonists and dual glucose-dependent insulinotropic polypeptide (GIP)/GLP-1 agonists on body composition. The analysis found that, in addition to significant reductions in total body weight and fat mass, ∼25 % of the total weight lost consisted of lean mass compared with control groups [22]. The magnitude of this loss with GLP-1 therapies has been likened to that typically accrued over a decade or more of aging [23]. Importantly, there was heterogeneity between agents: liraglutide (3.0 mg weekly and 1.8 mg daily) achieved weight loss without significant lean mass reduction, whereas tirzepatide (15 mg weekly) and semaglutide (2.4 mg weekly) were least effective in preserving lean mass [22]. These findings underscore the need for careful consideration when prescribing GLP-1 therapies, particularly in the context of sarcopenia, where losses in muscle quantity and function can have significant clinical consequences.

The clinical consequences of this lean mass loss depend on context. In younger, otherwise healthy individuals, muscle quality may improve due to reductions in intramuscular fat, often without measurable loss in strength. However, in older adults with co-morbidities or those with sarcopenic obesity, even modest reductions in lean mass can exacerbate functional decline [24]. Weight cycling, a scenario likely in real-world GLP-1 therapy use due to treatment discontinuation and subsequent weight regain, further increases this risk, as repeated cycles of weight loss and regain are associated with cumulative muscle loss and progressive declines strength [25].

Given these concerns, targeted strategies integrating nutrition and physical activity are essential to mitigate lean mass loss during GLP-1 therapy use and to preserve muscle health and function. In older adults, higher protein intake is consistently associated with better preservation of lean mass. The Health ABC Study, a three-year longitudinal analysis in 2066 community-dwelling older adults aged 70–79, found that those in the highest quintile of protein intake (∼18.2 % of total energy) lost nearly 40 % less lean mass than those in the lowest quintile (<12.7 % of total energy intake), even after adjusting for confounders [24]. A systematic review and meta-analysis reported that total protein intakes greater than 1.2–1.6 g/kg/day, when combined with resistance training, may result in small additional gains in lean body mass compared with lower intakes in adults over 65 years of age [26]. These findings were derived from studies conducted in populations not receiving GLP-1 therapies and therefore provide a physiological rationale supporting the role of higher protein intake in preserving muscle mass, though direct evidence in individuals treated with GLP-1 therapies remains lacking. Similarly, in a randomized controlled trial involving young adults undergoing marked energy restriction (∼40 % calorie deficit) and resistance exercise training, a higher-protein diet (2.4 g/kg/day) led to greater increases in lean body mass and greater fat mass loss compared with a lower-protein diet (1.2 g/kg/day), which produced minimal lean mass change and smaller fat loss [27]. Timing of protein intake may also matter. A 7-day crossover feeding study showed that distributing protein evenly across meals (∼25–30 g per breakfast, lunch, and dinner) increased muscle protein synthesis by 25 % compared with a skewed intake pattern [28,29]. Together, these findings support a dietary strategy of consuming >1.2 g/kg/day of protein, evenly distributed across meals, alongside resistance training to help preserve lean mass during GLP-1 therapy.

The S-LiTE trial provides the most direct clinical evidence supporting the role of exercise in lean mass retention during GLP-1 therapy. The intervention involved supervised, group-based aerobic and resistance training totaling more than 150 minutes per week at 60–85 % of maximum heart rate [6]. When combined with liraglutide, this structured exercise program produced the greatest total weight loss among all treatment arms, primarily through increased fat mass reduction while preserving lean mass. In contrast, liraglutide alone maintained overall weight loss but was associated with loss of both fat and lean tissue [6]. These findings emphasize that incorporating structured exercise into GLP-1 therapy is important for maintaining muscle mass and functional health during weight loss.

## 5 Managing gastrointestinal side-effects and preventing treatment discontinuation with GLP-1 therapies

GI symptoms are the most common adverse effects of GLP-1 therapies, with nausea, constipation, diarrhea, and vomiting occurring in around 40 % of patients [11,[30], [31], [32]]. Other clinically relevant GI complications include cholelithiasis, which occurred about 50 % more often, and gastroesophageal reflux disease (GERD), which occurred roughly twice as often, in patients receiving GLP-1 therapies compared with placebo [33]. Because GI symptoms are the leading cause of treatment discontinuation [10,12], implementing preventive and management strategies is essential to maximize adherence and therapeutic benefit.

Symptom-specific management can improve tolerance and reduce treatment discontinuation. For nausea and vomiting, dietary adjustments may include smaller, more frequent meals and avoidance of high-fat or spicy foods, which can exacerbate symptoms [34,35]. Gradual dose titration of GLP-1 therapies can also improve tolerability, typically involving dose escalation every 4 weeks or longer, with temporary maintenance or dose reduction when adverse effects occur [34,35]. If symptoms persist, prophylactic use of antiemetics may be considered based on individual symptom severity and regional drug availability. However, robust clinical data directly comparing the safety and efficacy of different antiemetic options in the context of GLP-1 therapies remain limited. Constipation may be mitigated by increasing dietary fiber (e.g., total intake of 14g/1000kcals/day) and fluid intake (>2–3 L/day), as well as to consider the use of stool softeners [34]. Diarrhea management includes increasing soluble fiber intake for bulking stool while avoiding dairy, caffeine, artificial sweeteners and high-sugar and high-fat foods [34,35].

Gallbladder-related risks are heightened during rapid weight loss, as demonstrated in trials of GLP-1 therapies and in dietary interventions such as very-low-calorie diets [33,36]. Measures such as, maintaining healthy dietary fat intake (e.g., 19–30 % of total energy intake; 1 tsp olive oil daily) may help for preventing gallstones [37]. Exacerbation of GERD with initial use of GLP-1 therapies may be managed with short-term use of proton pump inhibitors or H2 receptor antagonists, noting that weight reduction achieved with GLP-1 therapies over the long-term often leads to improvement in GERD symptoms [34,35].

## 6 Future directions and research gaps

Although GLP-1 therapies have revolutionized obesity and diabetes management, important knowledge gaps remain in optimizing their long-term safety, tolerability, and body composition outcomes.

From a nutrition and lifestyle standpoint, key unanswered questions include how these agents influence eating patterns, diet quality, and macro- and micronutrient intake over time, and whether specific dietary changes, particularly in protein intake, affect body composition during weight loss with GLP-1 therapies. Establishing evidence-based dietary guidance tailored to users of GLP-1 therapies is an urgent priority, especially for sustaining muscle mass and function.

In the context of lean mass preservation, future research should integrate more precise and clinically meaningful measures of muscle health, including muscle composition, mobility, and strength, rather than relying solely on body composition changes. Ongoing pharmacologic developments, such as activin receptor antagonists (e.g., bimagrumab) [38], show early promise for selectively increasing lean mass while reducing fat mass. However, evidence remains preliminary, and their long-term efficacy and safety, particularly in combination with GLP-1 therapies, have yet to be established. Until such strategies are validated, structured resistance training and adequate protein intake (>1.2 g/kg/day) remain the most practical, evidence-based approaches, especially for older adults, sarcopenic patients, or individuals at elevated risk of functional decline.

In terms of tolerability, GI side effects remain the leading cause of treatment discontinuation, yet optimal prevention and management strategies require further refinement. Research should address whether personalized titration schedules, dietary modifications, or adjunctive pharmacologic agents can improve adherence without compromising efficacy. Moreover, rare or poorly understood adverse effects such as, sulfur burps (eructation with hydrogen sulfide odor) warrant mechanistic investigation. Hypotheses include medication-induced delayed gastric emptying, small intestinal bacterial overgrowth, and dietary sulfur load, but direct evidence is lacking. Clarifying these pathways may guide preventive or therapeutic strategies and improve patient quality of life.

## 7 Conclusion

• Dietary modification personalized to individual preferences and treatment goals (e.g., caloric deficits of ∼500 kcal/day), combined with structured physical activity (e.g., ≥150 minutes of moderate-to-vigorous exercise per week), produces additive benefits beyond GLP-1 therapy alone, enhancing weight loss, improving body composition, and reducing visceral fat and inflammation.
• Targeted strategies integrating nutrition (e.g., dietary intake of >1.2 g/kg/day of protein distributed evenly across meals), and physical activity (e.g., regular resistance training) are essential to mitigating lean mass loss associated with some GLP-1 therapies.
• Gastrointestinal (GI) symptoms such as nausea, vomiting, constipation, and diarrhea are the most common adverse effects of GLP-1 therapies and are a leading cause of treatment discontinuation. Management strategies include gradual dose adjustment, increasing dietary fiber intake (approximately 14 g per 1000 kcal/day), maintaining adequate hydration (>2–3 L of fluid per day), and, when necessary, including short-term use of prophylactic antiemetics to support adherence and tolerability.

## Author contributions

JCN drafted the manuscript. LVG, IN, AF, AFHP, LC, CWCK, and JLS reviewed the manuscript for important intellectual content. All authors reviewed and approved the final manuscript.

## Declaration of artificial intelligence (AI) and AI-assisted technologies

During the preparation of this work, the authors used ChatGPT to generate the graphical abstract. After using ChatGPT, the authors reviewed and edited the content as needed and take full responsibility for the content of the publication.

## Funding

This work was supported by an unrestricted educational grant from Nestle Health Sciences through the Toronto 3D Knowledge Synthesis and Clinical Trials foundation. The sponsors had no involvement in any aspect of this review, including its conception, literature selection, analysis, manuscript preparation, review, approval, or the decision to publish.

## Conflicts of interest

JCN was supported by a Toronto 3D Scholarship.

LVG is a member of the Speakers Bureau for Bayer Pharma, Boehringer Ingelheim, Eli Lilly & Co, Nestlé Health Science, Novo Nordisk, and Regeneron Pharma.

IN has served as a speaker, consultant, and/or advisory board member for Boehringer Ingelheim, Eli Lilly, Bayer Pharmaceuticals, MJH Life Sciences, and Dexcom, Inc. IN has also received grant support from the National Institutes of Health (US), the American Heart Association, Novartis, and Amgen.

AF has served as an advisor and/or speaker for Eli Lilly, Novo Nordisk, Boehringer Ingelheim, Abbott, AbbVie, Amgen, Samsung, Metsera, and Currax, and as a speaker for Rhythm and Nestlé. AF has also received research funding from Eli Lilly and Viking.

LC has received research support from the Canadian Institutes of Health Research (CIHR), Protein Industries Canada (a Government of Canada Global Innovation Clusters), Alberta Pulse Growers, and The United Soybean Board (USDA soy “Checkoff” program).

CWCK has received grants or research support from the Advanced Food Materials Network, Agriculture and Agri-Foods Canada (AAFC), Almond Board of California, Barilla, Canadian Institutes of Health Research (CIHR), Canola Council of Canada, International Nut and Dried Fruit Council, International Tree Nut Council Research and Education Foundation, Loblaw Brands Ltd, the Peanut Institute, Pulse Canada, and Unilever. He has received in-kind research support from the Almond Board of California, Barilla, California Walnut Commission, Kellogg Canada, Loblaw Companies, Nutrartis, Quaker (PepsiCo), the Peanut Institute, Primo, Unico, Unilever, and WhiteWave Foods/Danone. He has received travel support and/or honoraria from the Barilla, California Walnut Commission, Canola Council of Canada, General Mills, International Nut and Dried Fruit Council, International Pasta Organization, Lantmannen, Loblaw Brands Ltd., Nutrition Foundation of Italy, Oldways Preservation Trust, Paramount Farms, the Peanut Institute, Pulse Canada, Sun-Maid, Tate & Lyle, Unilever, and White Wave Foods/Danone. He has served on the scientific advisory board for the International Tree Nut Council, International Pasta Organization, McCormick Science Institute, and Oldways Preservation Trust. He is a founding member of the International Carbohydrate Quality Consortium (ICQC), Chair of the Diabetes and Nutrition Study Group (DNSG) of the European Association for the Study of Diabetes (EASD), and is a Director of the Toronto 3D Knowledge Synthesis and Clinical Trials foundation.

JLS has received research support from the Canadian Foundation for Innovation, Ontario Research Fund, Province of Ontario Ministry of Research and Innovation and Science, Canadian Institutes of health Research (CIHR), Diabetes Canada, American Society for Nutrition (ASN), International Nut and Dried Fruit Council (INC) Foundation, National Honey Board (U.S. Department of Agriculture [USDA] honey “Checkoff” program), Institute for the Advancement of Food and Nutrition Sciences (IAFNS; formerly ILSI North America), Pulse Canada, Quaker Oats Center of Excellence, The United Soybean Board (USDA soy “Checkoff” program), Protein Industries Canada (a Government of Canada Global Innovation Clusters), The Tate and Lyle Nutritional Research Fund at the University of Toronto, The Glycemic Control and Cardiovascular Disease in Type 2 Diabetes Fund at the University of Toronto (a fund established by the Alberta Pulse Growers), The Plant Protein Fund at the University of Toronto (a fund which has received contributions from IFF), and The Nutrition Trialists Network Research Fund at the University of Toronto (a fund which has received donations from the Calorie Control Council, Physicians Committee for Responsible Medicine, and vegan grants through the Karuna Foundation). He has received food donations to support randomized controlled trials from the Almond Board of California, California Walnut Commission, Peanut Institute, Barilla, Unilever/Upfield, Unico/Primo, Loblaw Companies, Quaker, Kellogg Canada, Danone, Nutrartis, Soylent, and Dairy Farmers of Canada. He has received travel support, speaker fees and/or honoraria from ASN, Danone, Dairy Farmers of Canada, FoodMinds LLC, Nestlé, Abbott, General Mills, Nutrition Communications, International Food Information Council (IFIC), Calorie Control Council, International Sweeteners Association, International Glutamate Technical Committee, Arab Beverages Association, and Phynova. He has or has had ad hoc consulting arrangements with Perkins Coie LLP, Tate & Lyle, Inquis Clinical Research, Ingredion, and Brightseed. He is a former member of the European Fruit Juice Association Scientific Expert Panel and a former member of the Soy Nutrition Institute (SNI) Scientific Advisory Committee. He is on the Clinical Practice Guidelines Expert Committees of Diabetes Canada, European Association for the study of Diabetes (EASD), Canadian Cardiovascular Society (CCS), and Obesity Canada/Canadian Association of Bariatric Physicians and Surgeons. He serves as an unpaid member of the Board of Trustees of IAFNS and formerly served as an unpaid scientific advisor for the Carbohydrates Committee of IAFNS. He is a Director at Large of the Canadian Nutrition Society (CNS), a founding member of the International Carbohydrate Quality Consortium (ICQC), an Executive Board Member of the Diabetes and Nutrition Study Group (DNSG) of the EASD, and a director of the Toronto 3D Knowledge Synthesis and Clinical Trials foundation. His spouse is an employee of AB InBev.

AFHP has no conflicts of interest to declare.
